# Quantitative and qualitative assessment of plasma cell dyscrasias in dual-layer spectral CT

**DOI:** 10.1007/s00330-021-07821-0

**Published:** 2021-03-30

**Authors:** S. C. Brandelik, S. Skornitzke, T. Mokry, S. Sauer, W. Stiller, J. Nattenmüller, H. U. Kauczor, T. F. Weber, T. D. Do

**Affiliations:** 1 Clinic of Diagnostic and Interventional Radiology (DIR), Heidelberg University Hospital, Im Neuenheimer Feld 420, 69120 Heidelberg, Germany; 2 Medical Department V, Hematology/Oncology/Rheumatology, Heidelberg University Hospital, Im Neuenheimer Feld 410, 69120 Heidelberg, Germany

**Keywords:** Computed tomography, Multiple myeloma, Plasma cell dyscrasia, Virtual non-calcium, Dual-layer spectral CT

## Abstract

**Objectives:**

Virtual non-calcium (VNCa) images could improve assessment of plasma cell dyscrasias by enhancing visibility of bone marrow. Thus, VNCa images from dual-layer spectral CT (DLCT) were evaluated at different calcium suppression (CaSupp) indices, correlating results with apparent diffusion coefficient (ADC) values from MRI.

**Methods:**

Thirty-two patients with initial clinical diagnosis of a plasma cell dyscrasia before any chemotherapeutic treatment, who had undergone whole-body low-dose DLCT and MRI within 2 months, were retrospectively enrolled. VNCa images with CaSupp indices ranging from 25 to 95 in steps of 10, conventional CT images, and ADC maps were quantitatively analyzed using region-of-interests in the vertebral bodies C7, T12, L1-L5, and the iliac bone. Independent two-sample *t*-test, Wilcoxon-signed-rank test, Pearson’s correlation, and ROC analysis were performed.

**Results:**

Eighteen patients had a non-diffuse, 14 a diffuse infiltration in conventional MRI. A significant difference between diffuse and non-diffuse infiltration was shown for VNCa-CT with CaSupp indices from 55 to 95, for conventional CT, and for ADC (each *p* < 0.0001). Significant quantitative correlation between VNCa-CT and MRI could be found with strongest correlation at CaSupp index 65 for L3 (*r* = 0.68, *p* < 0.0001) and averaged L1-L5 (*r* = 0.66, *p* < 0.0001). The optimum CT number cut-off point for differentiation between diffuse and non-diffuse infiltration at CaSupp index 65 for averaged L1-L5 was −1.6 HU (sensitivity 78.6%, specificity 75.0%).

**Conclusion:**

Measurements in VNCa-CT showed the highest correlation with ADC at CaSupp index 65. VNCa technique may prove useful for evaluation of bone marrow infiltration if MRI is not feasible.

**Key Points:**

*• VNCa-CT images can support the evaluation of bone marrow infiltration in plasma cell dyscrasias.*

*• VNCa measurements of vertebral bodies show significant correlation with ADC in MRI.*

*• Averaging L1-L5 at CaSupp index 65 allowed quantitative detection of infiltration comparable to MRI ADC.*

## Introduction

Multiple myeloma (MM) and its precursors monoclonal gammopathy of undetermined significance (MGUS) and smoldering myeloma (SMM) are characterized by monoclonal proliferation of plasma cells with the spine as the predominant site of active hematopoietic bone marrow being the primary target. For MM, different infiltration patterns of the bone marrow have been described in conventional MRI [1]—normal, focal, diffuse, mixed, and variegated salt-and-pepper pattern—and were shown to have a prognostic impact [2, 3]. Diffusion-weighted imaging sequences (DWIs) and the apparent diffusion coefficient (ADC) in MRI have been shown to correlate with cellularity of the bone marrow in MM [4, 5]. ADC values differ significantly between infiltration patterns [6]. The International Myeloma Working Group currently recommends whole-body low-dose CT as the imaging modality of choice for the initial assessment in monoclonal plasma cell diseases based on the importance of detection of osteolytic lesions for disease definition and detection of imminent disabling fractures [7, 8]. Only in certain cases, such as equivocal CT results in MGUS and inconclusive CT in suspected SMM and MM, MRI is currently recommended as a further imaging modality [7]. However, particularly diffuse hypercellularity is obscured on conventional CT, and to date, MRI is the best non-invasive modality to detect bone marrow infiltration which is not necessarily associated with osteolytic bone destruction [7, 9].

In dual-layer spectral CT (DLCT), two different detector layers atop each other absorb different parts of the polychromatic patient-attenuated X-ray spectrum instead of activating a second X-ray tube or rapid-voltage switching tube before performing the examination. The benefit is the opportunity of retrospective spectral analyses and the selective depiction or suppression of materials, e.g., uric acid [10], iodine [11], or calcium [12]. In virtual non-calcium (VNCa) images, the osseous component is removed from the spectral base data in order to improve visualization of bone marrow. The degree of calcium suppression depends on the calcium suppression (CaSupp) index, which defines the calcium composition level.

Using the dual-source dual-energy technique, significantly increased VNCa-CT numbers in MRI-confirmed bone marrow lesions compared to non-infiltrated bone marrow were found [13] and VNCa-CT numbers differed significantly between different infiltration patterns [14]. The aim of this study is to correlate conventional and VNCa-CT images with MRI images to evaluate the quantitative and qualitative assessment of bone marrow infiltration in DLCT compared to the gold standard MRI.

## Material and methods

### Ethics approval and consent

This retrospective exploratory single-center study was approved by the local review board. The need for written informed consent was waived.

### Clinical data selection and study design

Patients were screened in the hospital and radiological information system (I.S.-H.*med., SAP; Centricity RIS-i, GE Healthcare) for study inclusion. Patients with an initial diagnosis of a plasma cell dyscrasia (MM, SMM or MGUS) and routinely performed whole-body low-dose DLCT and whole-body MRI within 2 months of each other from August 2018 until October 2019 were enrolled into the study. Exclusion criteria were prior anti-myeloma chemotherapeutic treatment, additional solid malignancies, extensive imaging artifacts, or missing spectral data.

The patient cohort consisted of 38 patients matching the inclusion criteria, of whom 6 patients were excluded due to missing spectral information. A *diffuse* infiltration of the axial bone marrow was present in 14 cases and a *non-diffuse* infiltration in 18 cases. Twenty-seven patients were diagnosed with MM (14 with *diffuse* infiltration and 13 with *non-diffuse* infiltration), 4 patients with SMM (all with *non-diffuse* infiltration), and 1 patient with MGUS (*non-diffuse* infiltration) (Table 1). Median age at time of CT was 62.5 years (range 38–78). Median time between CT and MRI was 2.5 days (range 0–50).

**Table 1:** Study population with type of plasma cell dyscrasia and infiltration

Plasma cell dyscrasia type	Number of patients
MGUS	1
MGUS diffuse infiltration	0
MGUS non-diffuse infiltration	1
Smoldering myeloma	4
Smoldering myeloma diffuse infiltration	0
Smoldering myeloma non-diffuse infiltration	4
Multiple myeloma	27
Multiple myeloma diffuse infiltration	14
Multiple myeloma non-diffuse infiltration	13

### DLCT acquisition and post-processing

Non-contrast CT acquisitions were performed as helical scans from the vertex of the skull to the knees using a dual-layer detector technique (IQon Spectral CT, Philips) with the following acquisition parameters: tube voltage 120 kV_p_, dose right index 15 (automated, attenuation-based dose modulation), average tube current-time product 93 mAs, mean computed tomography dose index (CTDI_vol_) 6.5 mGy (standard deviation [SD] 0.7 mGy, range 5.1–7.6 mGy, 32 cm body-phantom), pitch 1.0, gantry rotation time of 0.75 s, and collimation 64 × 0.625 mm. The field of view (FOV) was variable depending on patient body volume.

VNCa image reconstructions of DLCT data and CT number measurements were performed with the manufacturer’s dedicated image post-processing software (IntelliSpace Portal Version 11, Philips). VNCa images were reconstructed with CaSupp indices ranging from 25 to 95 in steps of 10 with minimum visibility of bony structures at index 25 and maximum visibility at index 95. Reconstruction was performed with slice thickness 3 mm, increment 1.5 mm, sharp kernel YA, and iterative reconstruction with iDose level 2 for conventional images and kernel B for VNCa images.

Using the same software, regions of interest (ROIs) were positioned manually in the vertebral bodies C7, Th12, L1-L5, and the right iliac bone to measure the respective CT numbers (mean value and SD). ROI sizes were 50 mm^2^ in C7 and 150 mm^2^ in all other locations (± 5 mm^2^). Inclusion of focal lesions into the ROI was avoided, if visible on CT or MRI. Pretreated vertebrae—radiotherapy, kyphoplasty, or spondylodesis—and adjacent vertebrae with treatment-associated artifacts or with unavoidable measurement of sclerosis were excluded. To ensure comparability, ROIs were copied between different CaSupp indices and conventional images.

A contrast-to-noise ratio (CNR) was calculated for each measurement location and for each CaSupp index:

$$ \mathrm{CNR}=\frac{\mid {\mu}_{\mathrm{diffuse}}-{\mu}_{\mathrm{non}-\mathrm{diffuse}}\mid }{\sigma } $$

Here, *μ*_non − diffuse_ is the mean CT number of all patients with non-diffuse infiltration, *μ*_diffuse_ is the mean CT number of all patients with diffuse infiltration, and *σ* is the mean of all measured SDs for the measurement location and the CaSupp index.

### MRI acquisition and analysis

Whole-body MRI was performed on a 1.5 Tesla MRI (MAGNETOM AvantoFit, Siemens Healthineers) with the following imaging protocol: unenhanced coronal T1-weighted turbo spin echo sequences, short tau inversion recovery (STIR) sequences, and transversal DWI sequences (*b*-values 50 and 800 s/mm^2^, five blocks of 46 × 46 cm) with calculation of ADC maps. A signal loss in T1 and concurrent signal increase in STIR was defined as plasma cell infiltration of the bone marrow—either with signal alteration of focal areas within a normal fat signal or with diffuse homogeneous signal alteration of the spine [1]. By means of a consensus read by all radiologists, the infiltration pattern of each patient was considered either as *diffuse* (defined as diffuse infiltration, variegated salt-and-pepper pattern [1] or intense disseminated focal infiltration) or as *non-diffuse* (defined as normal pattern or sporadic focal infiltration). T1 and STIR sequences were also screened to avoid inclusion of large focal lesions into the quantitative evaluation. The consensus read was considered standard of reference for this study.

In the institutional PACS (GE Healthcare), ROIs were positioned manually in the ADC maps of the vertebral bodies C7, Th12, L1-L5, and the right iliac bone to obtain the respective mean signal intensity (ADC value) and SD. ROI sizes were 50 mm^2^ in C7, 200–250 mm^2^ in Th12, L1-L5, and 100–150 mm^2^ in the iliac bone (± 5 mm^2^). Slice thickness was 5 mm. Inclusion of focal lesions was avoided if determinable. CNR for each measurement location and CaSupp index was calculated for ADC values as described above for CT numbers.

### Image analysis

Quantitative and qualitative image analysis was performed independently by two radiologists (4 and 8 years of experience).

**Fig. 1:**
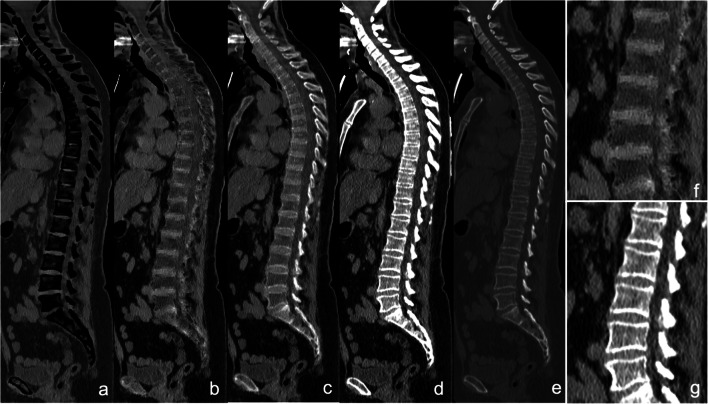
Patient with non-diffuse infiltration. **a** CaSupp index 25; **b** CaSupp index 65; **c** CaSupp index 95; **d** conventional CT soft tissue window; **e** conventional CT bone window; **f**, **g** magnification of **b** and **d** for L1-5

**Fig. 2:**
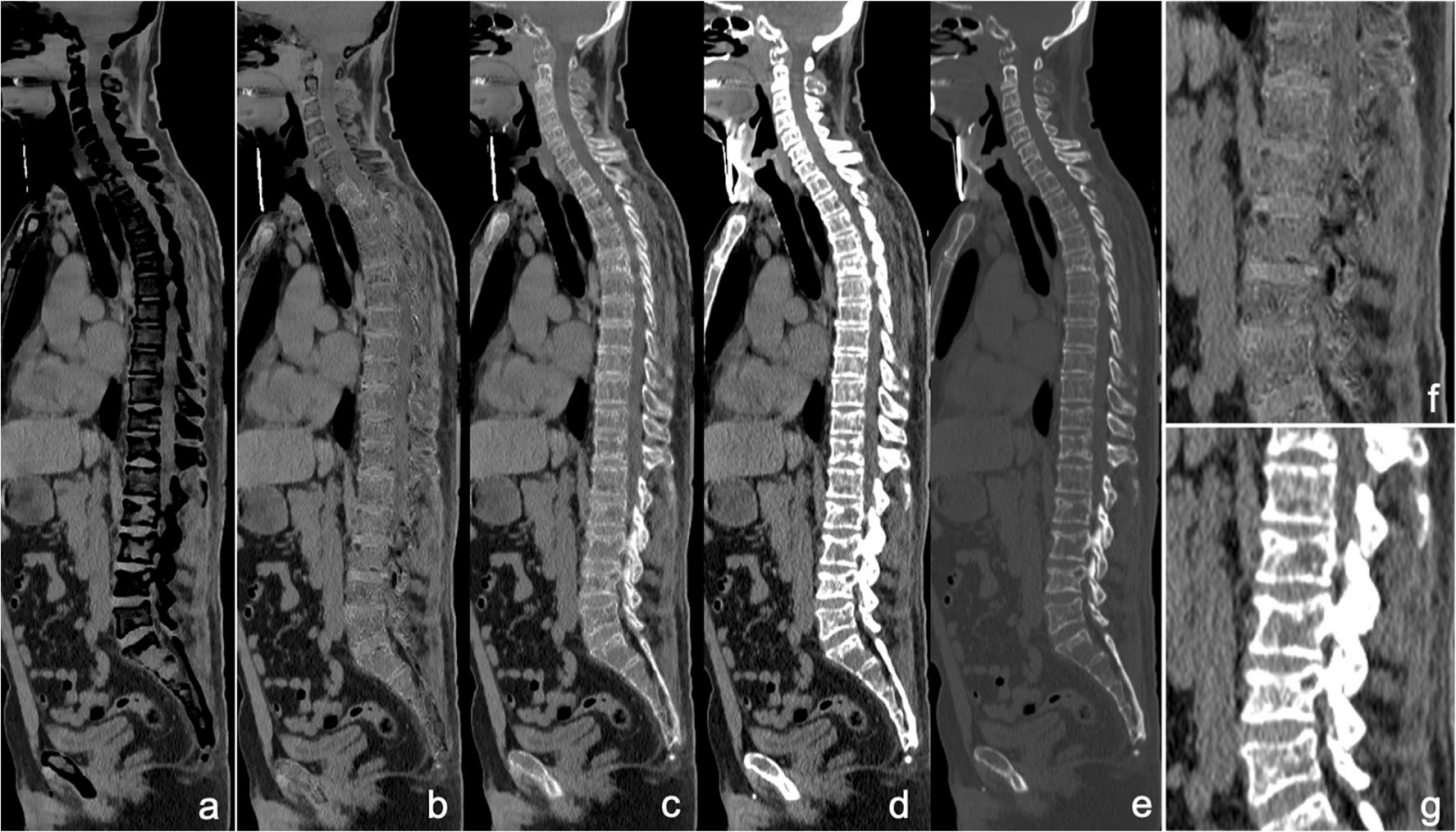
Patient with diffuse infiltration; **a** CaSupp index 25; **b** CaSupp index 65; **c** CaSupp index 95; **d** conventional CT soft tissue kernel; **e** conventional CT sharp kernel; **f**, **g** magnification of **b** and **d** for L1-5

For qualitative assessment of VnCa images, areas within the bone marrow that visually showed a density comparable to that of the erector spinae or gluteal muscles were defined as areas with plasma cell infiltration. Using the same criteria as described above for MRI, a decision was made for *non-diffuse* (Fig. 1) or *diffuse* infiltration (Fig. 2) on VNCa images at CaSupp index 65, which showed the strongest correlation to ADC in the quantitative evaluation.

### Statistical analysis

Statistical analysis was performed using SAS Version 9.4 (SAS Institute Inc.). Descriptive statistics were calculated, determining means and SDs for normal distributed data. For assessment of significant differences in CT numbers between the infiltration patterns, an independent two-sample *t*-test was applied (conventional and VNCa-CT) following a normal distribution. For MRI, a Wilcoxon-signed-rank test was applied for the same question (non-normal distribution of data). Correlations between VNCa-CT numbers and ADC values were calculated using Pearson’s correlation. Receiver operating characteristic (ROC) analysis with calculation of the area under the ROC curve (AUC) and Youden’s J statistic were performed to determine the cut-off for the mean VNCa-CT number of L1-L5 and of L3 at CaSupp index 65, which showed the highest correlation to ADC. AUCs were compared with each other using a large-sample *χ*^2^ test. The intraclass correlation coefficient (ICC) was calculated for determination of the interrater reliability of the quantitative and qualitative assessment. The significance level for statistical testing was set at *p* < 0.05.

## Results

### Quantitative analysis

Table 2 shows CT numbers and ADC values of different vertebral bodies, infiltration types, and CaSupp indices. CT numbers in VNCa images were significantly different between *diffuse* and *non-diffuse* infiltration at CaSupp indices from 55 to 95 (each with *p* < 0.0001). CT numbers in conventional images as well as ADC values also showed significant differences between both groups (both *p* < 0.0001).

**Table 2:** Means and standard deviations of MRI ADC and CT numbers in conventional and VNCa images for all measurement locations, splitted into diffuse and non-diffuse infiltration pattern.

Location	Infiltration pattern	MRI ADC	CT conventional	CaSupp 25	CaSupp 35	CaSupp 45	CaSupp 55	CaSupp 65	CaSupp 75	CaSupp 85	CaSupp 95
		[AU]	[HU]	[HU]	[HU]	[HU]	[HU]	[HU]	[HU]	[HU]	[HU]
C7	Diffuse	754.9 ± 248.8	243.3 ± 75.4	−278.3 ± 124.1	− 153.8 ± 73.0	−78.2 ± 48.7	−27.9 ± 34.6	8.2 ± 27.2	35.6 ± 24.6	58.5 ± 25.7	78.1 ± 28.1
	Non-diffuse	496.1 ± 143.7	132.9 ± 36.2	−159.8 ± 49.9	−86.8 ± 31.3	−45.2 ± 22.1	−17.9 ± 17.6	2.2 ± 15.7	17.0 ± 15.6	29.4 ± 16.3	39.7 ± 17.4
T12	Diffuse	681.2 ± 352.9	155.3 ± 56.3	−151.3 ± 66.2	−75.6 ± 37.4	−32.4 ± 21.8	−4.0 ± 13.5	16.5 ± 11.0	32.3 ± 12.7	45.1 ± 15.8	55.9 ± 19.1
	Non-diffuse	520.7 ± 16..8	218.5 ± 74.6	−275.9 ± 128.9	−149.1 ± 80.2	−80.6 ± 54.8	−33.9 ± 39.6	−0.25 ± 31.1	25.3 ± 27.4	46.6 ± 27.1	63.7 ± 28.9
L1	Diffuse	655.7 ± 265.6	150.6 ± 59.0	−152.1 ± 70.1	−76.7 ± 40.3	−34.0 ± 24.4	−5.9 ± 15.8	14.5 ± 12.8	30.1 ± 13.8	43.0 ± 15.8	53.4 ± 19.4
	Non-diffuse	419.9 ± 108.2	120.5 ± 39.2	−142.9 ± 47.0	−77.8 ± 28.4	−40.7 ± 19.2	−16.1 ± 15.2	1.3 ± 14.0	14.9 ± 14.6	25.9 ± 15.9	35.1 ± 17.4
L2	Diffuse	660.5 ± 310.9	146.4 ± 44.3	−159.5 ± 59.2	−83.8 ± 35.9	−40.5 ± 23.9	−11.9 ± 17.7	8.6 ± 15.4	24.5 ± 15.4	37.3 ± 16.7	48.1 ± 18.5
	Non-diffuse	438.4 ± 109.8	116.8 ± 44.9	−144.1 ± 56.9	−78.9 ± 33.3	−42.6 ± 20.6	−18.3 ± 14.0	−0.8 ± 11.7	12.7 ± 12.4	23.6 ± 14.3	32.8 ± 16.6
L3	Diffuse	689.4 ± 307.2	137.5 ± 44.0	−141.6 ± 62.5	−71.8 ± 37.5	−32.2 ± 23.8	−6.2 ± 16.1	12.6 ± 12.5	27.1 ± 12.0	38.8 ± 13.4	48.7 ± 15.6
	Non-diffuse	437.2 ± 139.2	115.6 ± 45.8	−154.5 ± 59.1	−87.7 ± 34.3	−49.6 ± 21.0	−24.4 ± 13.8	−6.3 ± 11.1	7.7 ± 11.7	19.0 ± 13.7	28.5 ± 16.2
L4	Diffuse	699.5 ± 343.9	133.8 ± 39.3	−159.9 ± 44.7	−87.2 ± 25.8	−45.9 ± 16.1	−18.6 ± 11.7	1.1 ± 10.8	16.2 ± 11.9	28.4 ± 13.8	38.7 ± 15.7
	Non-diffuse	457.4 ± 174.1	121.7 ± 48.6	−160.5 ± 71.8	−91.5 ± 42.8	−52.3 ± 26.9	−26.5 ± 17.6	−7.9 ± 12.9	6.5 ± 12.1	18.1 ± 13.6	27.6 ± 15.9
L5	Diffuse	717.3 ± 330.3	147.0 ± 42.8	−182.5 ± 60.5	−99.3 ± 36.0	−52.3 ± 22.8	−21.4 ± 15.8	1.0 ± 13.3	18.2 ± 13.7	32.1 ± 15.5	43.8 ± 17.8
	Non-diffuse	432.3 ± 115.2	127.7 ± 53.1	−178.4 ± 80.7	−102.1 ± 47.7	−58.5 ± 29.5	−29.9 ± 18.8	−9.2 ± 13.3	6.7 ± 12.5	19.6 ± 14.5	30.4 ± 17.6
L1-L5	Diffuse	684.51 ± 301.91	143.06 ± 41.93	−159.09 ± 52.19	−83.78 ± 29.96	−40.97 ± 18.08	−12.80 ± 11.90	7.54 ± 10.08	23.19 ± 11.16	35.92 ± 13.18	46.56 ± 15.69
	Non-diffuse	437.06 ± 113.75	120.46 ± 44.48	−156.08 ± 58.29	−87.61 ± 33.50	−48.73 ± 20.04	−23.02 ± 12.52	−4.57 ± 9.59	9.69 ± 10.35	21.23 ± 12.65	30.90 ± 15.24
Iliac bone	Diffuse	789.0 ± 310.2	156.4 ± 79.8	−162.3 ± 102.4	−84.2 ± 56.8	−38.3 ± 32.0	−8.6 ± 17.8	12.8 ± 13.3	29.3 ± 16.8	42.5 ± 22.6	53.9 ± 28.0
	Non-diffuse	521.6 ± 125.5	104.2 ± 58.1	−129.6 ± 74.9	−73.7 ± 42.4	−41.8 ± 24.6	−20.4 ± 15.9	−5.7 ± 11.5	5.9 ± 13.3	15.3 ± 16.8	23.3 ± 20.5

**Fig. 3:**
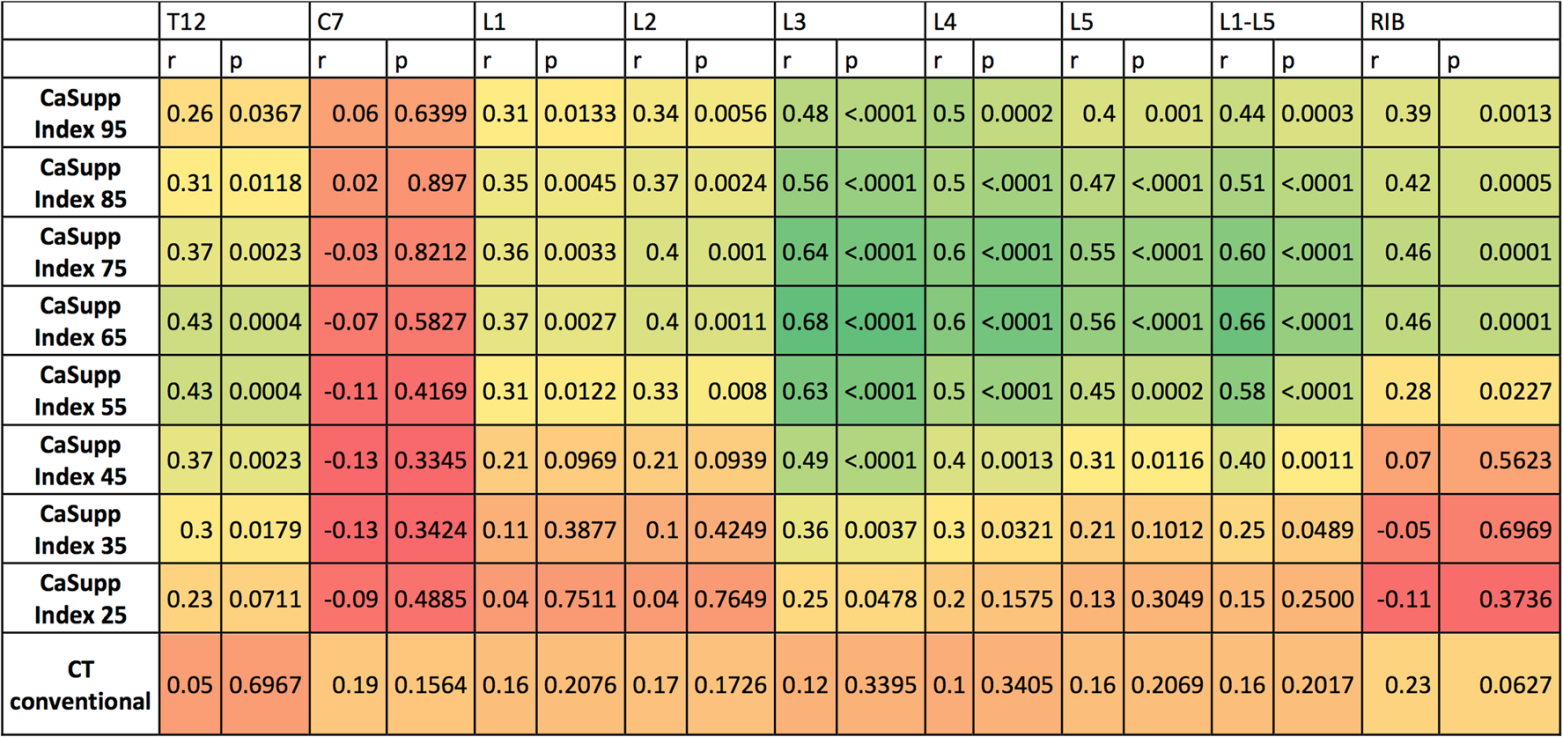
Heat map of Pearson’s correlation *r* and *p* value between CT numbers and ADC values for different CaSupp indices and measurement locations

Figure 3 summarizes the statistical results regarding the correlation between CT numbers and ADC values for different CaSupp indices and measurement locations. Regardless of the location, the highest correlation of VNCa-CT numbers and ADC values was at CaSupp indices from 55 to 95 (Pearson’s r between 0.26 and 0.68; all *p* < 0.05). Correlation of CT numbers in conventional images and ADC values was lower than that of CT numbers measured at CaSupp indices from 55 to 95 (Pearson’s *r* between 0.05 and 0.23; all *p* > 0.05). No significant correlation of CT numbers and ADC values could be shown in the vertebra C7 for all CaSupp indices. For each CaSupp index, the highest correlation of CT numbers and ADC values was in the vertebra L3. The strongest correlation of VNCa-CT numbers and ADC values for averaged L1-5 vertebrae (*r* = 0.66; *p* < 0.0001) and L3 vertebra (*r* = 0.68; *p* < 0.0001) was found at CaSupp index 65.

**Fig. 4:**
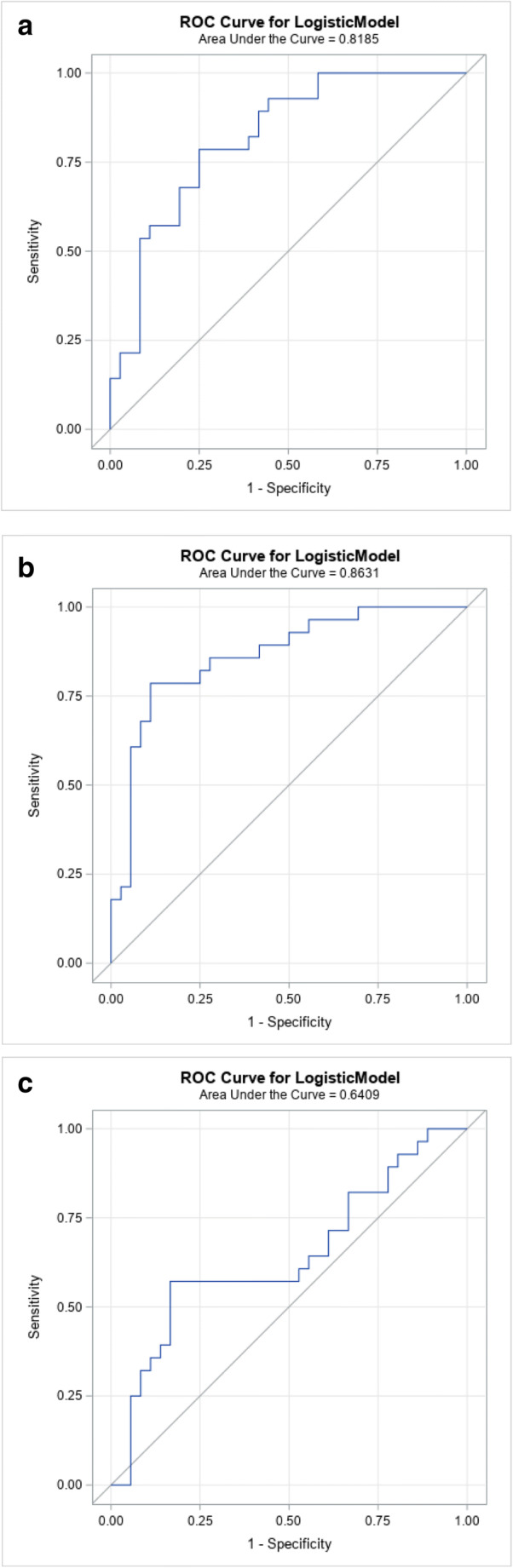
ROC curves for CT and MRI; **a** ROC curve analysis: good differentiation between diffuse and non-diffuse infiltration for mean L1-5 vertebrae at CaSupp index 65 with AUC of 0.8185; **b** ROC curve of the MRI ADC for mean of L1-5 vertebrae with AUC of 0.8631. **c** ROC curve of conventional CT for mean of L1-5 vertebrae with AUC of 0.6409

**Fig. 5:**
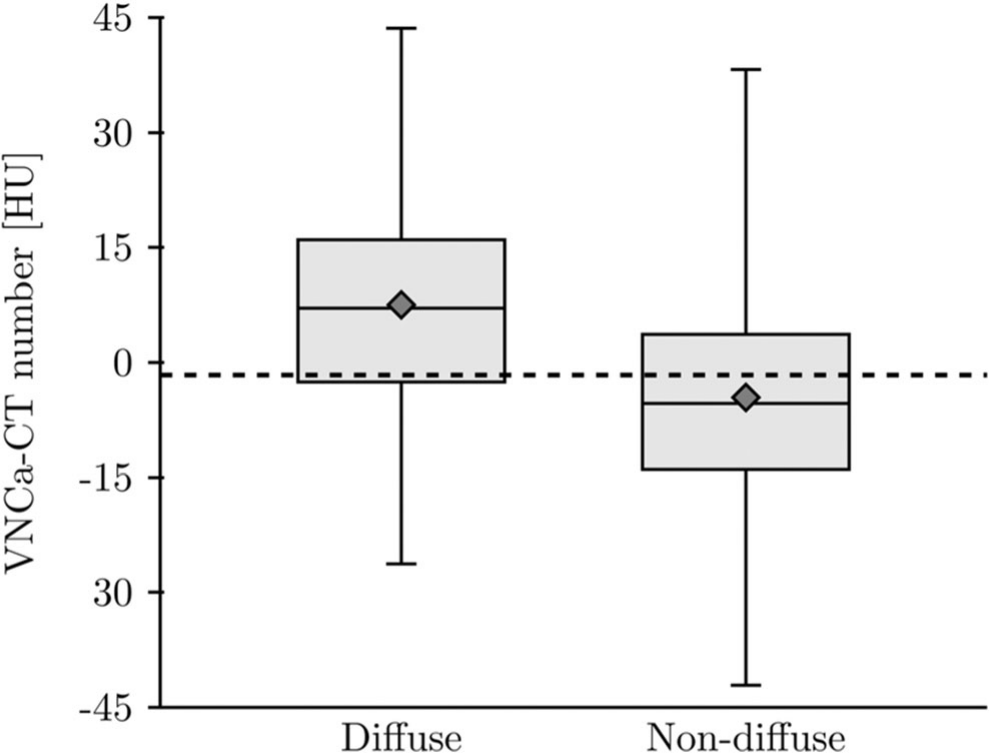
Boxplots for VNCa-CT numbers for diffuse and non-diffuse infiltration at CaSupp index 65 for mean of L1-5 vertebra. Dashed line: optimum cut-off point of −1.6 HU for differentiation between both patterns.

ROC analysis for the differentiation between *diffuse* and *non-diffuse* infiltration for mean values of L1-5 and L3 alone revealed similar AUCs for DLCT at CaSupp index 65 (AUC = 0.819 and AUC = 0.885) (Fig. 4a) and for ADC (AUC = 0.863 and AUC = 0.846) (Fig. 4b), but lower AUCs for conventional CT (AUC = 0.641 and AUC = 0.636) (Fig. 4c). For mean values of L1-5, the AUC for conventional CT was significantly below that for VNCa-CT with CaSupp index 65 (*p* = 0.048) and ADC (*p* = 0.01), while there was no significant difference in AUC between VNCa-CT with CaSupp index 65 and ADC (*p* = 0.526). The optimum cut-off point for VNCa-CT in L1-L5 was −1.6 HU, with higher values indicating a *diffuse* infiltration with a sensitivity of 78.6% and a specificity of 75.0% (Fig. 5). The cut-off for ADC was 487.6 AU with values above indicating a *diffuse* infiltration with a sensitivity of 78.6% and a specificity of 86.1%. Considering L3 alone, the optimum cut-off points were 3.1 HU for VNCa-CT (sensitivity: 78.6%, specificity 88.9%) and 516.7 AU for ADC (sensitivity: 75.0%, specificity 86.1%).

On average, CNR of VNCa images was highest in L3. For VNCa images, the CNR for CaSupp indices from 65 to 95 exceeded the CNR for conventional CT on average. For CaSupp indices from 65 to 95, the highest CNR was found in the iliac bone.

Table 3 summarizes the CNR for different locations and image data in conventional CT, VNCa images, and MRI.

**Table 3:** Contrast-to-noise ratio for different locations and image data in conventional CT, VNCa-CT, and MRI. *RIB* right iliac bone

	T12	C7	L1	L2	L3	L4	L5	L1-L5	RIB
Conventional CT	0.70	0.74	0.96	0.91	0.67	0.38	0.54	0.69	1.37
CaSupp index 25	0.25	−0.06	−0.28	−0.43	0.37	0.02	−0.11	−0.09	−0.79
CaSupp index 35	0.48	−0.17	0.05	−0.20	0.65	0.18	0.11	0.16	−0.40
CaSupp index 45	0.69	0.11	0.36	0.11	0.89	0.33	0.31	0.40	0.18
CaSupp index 55	0.85	0.35	0.62	0.36	1.07	0.45	0.47	0.59	0.73
CaSupp index 65	0.92	0.54	0.82	0.57	1.17	0.54	0.59	0.73	1.22
CaSupp index 75	0.97	0.68	0.95	0.72	1.20	0.59	0.67	0.82	1.52
CaSupp index 85	0.97	0.78	1.06	0.83	1.19	0.61	0.71	0.88	1.67
CaSupp index 95	0.96	0.88	1.09	0.90	1.18	0.64	0.73	0.90	1.76
MRI ADC	1.34	1.92	1.67	1.49	1.67	1.50	1.94	1.65	2.22

### Qualitative analysis

At CaSupp index 65, *diffuse* infiltration was detected with a sensitivity and specificity of 83.3% and 78.6% for reader 1 and 92.3% and 68.4% for reader 2. Accordingly, average sensitivity and specificity were 87.8% and 73.5% for qualitative analysis.

### Interrater reliability of quantitative and qualitative analysis

The interrater reliability of all quantitative measurements in CT was very high with an ICC of 0.98.

The interrater reliability of the qualitative assessment for the differentiation between *diffuse* versus *non-diffuse* infiltration in DLCT at CaSupp index 65 was substantial with an ICC of 0.69 [15].

## Discussion

VnCa imaging on DLCT providing spectral data gives the opportunity to detect osteolytic bone lesions and evaluate bone marrow involvement in a quantitative and qualitative manner [16]. Therefore, our study aimed for a detailed quantitative assessment of the performance of VNCa-DLCT in the evaluation of bone marrow infiltration in plasma cell dyscrasias compared to MRI, considering CaSupp index and location of measurement.

The results show that differentiating MRI-confirmed *diffuse* infiltration from *non-diffuse* infiltration in quantitative DLCT is possible at high CaSupp indices, also showing comparable ROC curves and AUCs to ADC measurements for VNCa-CT. Strong correlations to ADC suggest that bone marrow infiltration can best be assessed in VNCa images at CaSupp index 65. The threshold of −1.6 HU for the average from measurements in L1-L5 vertebrae can be used for differentiation between *diffuse* and *non-diffuse* infiltration, reaching a sensitivity of 78.6% and a specificity of 75.0%.

The highest correlation of CT and MRI in the L3 vertebra might be due to the fact that L3 has the largest distance to junctions between kyphosis and lordosis with less likely sclerosis/fractures. The poor results for C7 can be attributed to beam hardening artifacts in CT—which might be overcome with future photon-counting detector technique [17]—and to position-provoked oblique sectioning of the small C7 in transversal MRI slices.

For CT number cut-off calculation, we chose to focus on the mean of L1-5 due to the large size of those vertebrae with maximized reliable measurement avoiding sclerosis, fractures, or disc herniations. Although bone marrow involvement in plasma cell dyscrasias is quite heterogeneous, ROI-measurement of the whole lumbar spine in the best performing CaSupp index 65 might be as representative as possible for differentiating *diffuse* and *non-diffuse* infiltration, while possible to integrate into clinical routine with acceptable effort.

Using DSCT, Kosmala et al distinguished infiltrated from normal bone marrow with a sensitivity of 93.3% and a specificity of 92.4%, exceeding the values reported in this study. However, Kosmala et al defined infiltrated marrow independent from the intensity of infiltration and placed ROIs directly on MRI-confirmed focal lesions [14]. In contrast, the main focus of this study is to delineate *diffuse* infiltration in CT, comprising MRI-confirmed diffuse or intense disseminated focal infiltration. Differences between the DSCT study and this DLCT study are also reflected by differences in cut-off CT numbers (−44.9 HU versus −1.6 HU) [14].

High sensitivities and specificities of 97–100% were also reached by another recent DSCT study from Kosmala et al differentiating MRI-determined diffuse or focal infiltration patterns from a normal pattern with cut-offs of 35.7 HU and −31.9 HU after placing ROIs in L4-S1, Th12 and the ilium [13]. However, for the differentiation between diffuse and focal pattern, they reported a low sensitivity of 33% and specificity of 25%. Again, besides different DECT techniques, different approaches of marrow infiltration classification and sites of measurement have to be considered.

Regarding qualitative assessment of VNCa-CT, a sensitivity and specificity of 91.3% and 90.9% was reported for differentiating infiltrated from normal bone marrow using color-coded DSCT VNCa maps [14], compared to the sensitivity and specificity of 87.8% and 73.5% for differentiation between diffuse and non-diffuse infiltration using DLCT VNCa grayscale images in this study, again considering different definitions of infiltration patterns.

In another DSCT study, Thomas et al achieved a sensitivity of 75.0% and specificity of 82.4% for the qualitative differentiation of no infiltration versus high-grade infiltration with color-coded maps, but a sensitivity of just 40.0% and specificity of 85.7% for the differentiation between no infiltration versus moderate- and high-grade infiltration [18]. For detection of solid tumor metastasis, diagnostic performance can be improved using contrast agent and low CaSupp indices (25 to 50) [19]. However, contrast media are usually not administered to patients with monoclonal plasma cell diseases due to increased risk for renal insufficiency, dose aspects, and the lack of necessity for the detection of osteolysis.

Currently, conventional MRI is the gold standard for bone marrow evaluation, but ADC measurements have been recommended to be added to standard protocols [4, 7]. Our study revealed an ADC cut-off of 487.6 for the differentiation between *diffuse* and *non-diffuse* infiltration with a sensitivity of 78.6% and a specificity of 86.1%. Koutoulidis et al showed higher sensitivity and specificity (100% and 98%) for the differentiation of diffuse versus normal bone marrow in MRI [6]. However, aside from different categories of bone marrow involvement, a comparison of specific ADC cut-offs in different studies is still not feasible due to the influence of technical parameters and patient-specific factors [4].

In summary, this study shows an approach for quantitative assessment of bone marrow infiltration in plasma cell dyscrasias in DLCT but further studies have to be performed in order to refine this approach for clinical application. The additional evaluation of spinal bone marrow infiltration in routinely performed CT would be of value, especially for patients with contraindications to MRI. The main limitation of this study is that with the current technical level of visualization in DLCT, it was not feasible to separate the five different infiltration patterns as proposed for MRI [1]. Hence, the main objective was to distinguish widespread infiltration in DLCT from low/no infiltration which was implemented by integration of intense disseminated focal infiltration into the *diffuse* category and sporadic focal infiltration together with the normal pattern into the *non-diffuse* category. This is in contrast to other studies, which used different definitions of infiltration patterns. Furthermore, future studies should investigate comparability of CT and MRI data acquired by different scanners, imaging protocols, and post-processing tools.

## Conclusion

Quantitative and qualitative assessment of VNCa images in DLCT is feasible to determine the extent of bone marrow infiltration in plasma cell dyscrasias, especially when the gold standard MRI is not possible. Quantitative assessment of VNCa images produces comparable results to ADC of MRI at CaSupp index 65, averaging L1-L5 vertebrae, and ROC analysis suggests a cut-off of −1.6 HU for the differentiation between *diffuse* and *non-diffuse* infiltration.

## Abbreviations

AU: Arbitrary unit
CaSupp index: Calcium suppression index
CNR: Contrast-to-noise ratio
DLCT: Dual-layer spectral detector CT
FOV: Field of view
MGUS: Monoclonal gammopathy of undetermined significance
SD: Standard deviation
SMM: Smoldering multiple myeloma
VNCa: Virtual non-calcium
